# A Case Report on Delayed-Onset Diffuse Axonal Injury

**DOI:** 10.1097/MS9.0000000000003447

**Published:** 2025-05-30

**Authors:** Sonu Adhikari, Rupesh Raut, Dinuj Shrestha, Susmita Shah, Ayam Bhattarai, Prakash Bista

**Affiliations:** a Department of Neurosurgery, Patan Academy of Health Science, Lalitpur, Nepal; b Birtamode Nagar Hospital, Birtamode, Nepal

**Keywords:** case report, delayed onset, diffuse axonal injury

## Abstract

**Introduction:**

Diffuse axonal injury (DAI) is characterized by immediate loss of consciousness lasting for more than 6 hours following traumatic head injury. It can be classified into three grades based on the duration of coma, severity, and the presence of radiological or histological evidence of lesions in various parts of the brain.

**Case presentation:**

In this report, we present a case of a 20-year-old non-alcoholic male sustaining a head injury following a fall from a swing. On presentation, his Glasgow Coma Scale (GCS) was 15, which greatly fluctuated between 10 and 15 during hospitalization. MRI of the brain was suggestive of grade three DAI. Unusually, he went into a coma after 50 hours of admission. He was subsequently intubated and managed conservatively at our facility.

**Clinical discussion:**

DAI is one of the major causes of disability, post-traumatic coma, and persistent vegetative state following traumatic events. It is typically marked by an immediate loss of consciousness, resulting in only a few cases being recognized with delayed presentation. This case report exemplifies a unique and noteworthy instance of delayed onset DAI.

**Conclusion:**

DAI is often associated with traumatic brain injury (TBI), and it should always be suspected when the clinical presentation of the patient is significantly more severe than the initial computed tomography (CT) head findings. Maintaining a strong clinical suspicion and considering the possibility of unusual findings can facilitate prompt diagnosis and management of patients with DAI in clinical practice.

## Introduction

Diffuse axonal injury (DAI) is characterized by an immediate loss of consciousness lasting for more than 6 hours with or without significant findings on a computed tomography (CT) scan of the head^[1,2]^. DAI can be classified into mild, moderate, and severe categories based on the duration of the coma and the presence and absence of lesions in the cerebral cortex, brainstem, and corpus callosum^[1]^. Due to its poor prognosis, approximately 60% of patients with severe DAI do not survive, while 20% may develop a vegetative state^[1]^. High-velocity motor vehicle accidents are the leading cause of DAI^[3]^. The primary mechanism behind DAI involves accelerating and decelerating forces that create shearing effects on the white matter, leading to microhemorrhages at the gray-white junction^[3]^. Magnetic resonance imaging (MRI) is recognized as the superior diagnostic tool for DAI, particularly when used alongside the clinical presentations of the patient^[3]^. In this study, we present a rare case of delayed onset DAI that challenges the conventional understanding of this condition. This case report has been reported following the Surgical CAse REport (SCARE) criteria^[4]^.

**HIGHLIGHTS**

- Diffuse axonal injury (DAI) is characterized by immediate loss of consciousness lasting for more than 6 hours following traumatic head injury.
- It can be classified into three grades based on duration of coma, severity, and presence of radiological or histological lesions in various areas of the brain.
- The delayed presentation of the DAI is rarely described in the literature.
- DAI should always be suspected when the clinical findings are disproportionately more severe than the initial CT head findings following a traumatic event.

## Case presentation

A 20-year-old non-alcoholic male presented to the emergency department with altered consciousness following a fall from a swing. He also had one episode of generalized tonic-clonic seizure reported by a visitor before his presentation to the hospital. During the initial evaluation at the emergency department, he was oriented to time, place, and person. He had a Glasgow Coma Scale (GCS) of 15 (E4V5M6) with bilaterally reactive, regular, and equal pupils. His vitals were within normal limits with an oxygen saturation of 98% maintained in the room air. A complete neurological examination was performed with unremarkable findings. The cranial nerves were grossly intact, speech was normal, motor and sensory examination was normal, and reflexes were intact. But he was drowsy and occasionally agitated. An abrasion was seen over his right upper and lower limbs. Serial X-rays and extended-FAST scans were done to rule out trauma to other parts of the body and internal bleeding, respectively. Laboratory findings were within normal limits. The patient had no known medical conditions.

**Figure 1.**
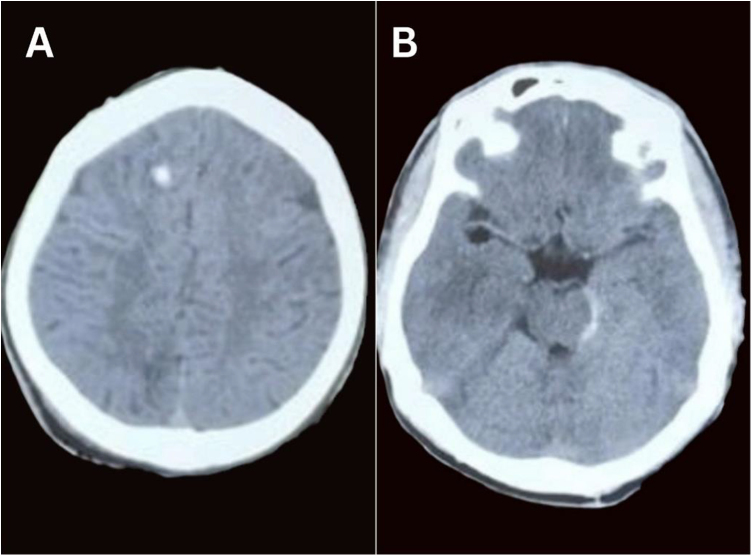
CT head showing hyperdense lesion in the right frontal region and subarachnoid hemorrhage over left peri mesencephalic cistern.

CT head was done at the emergency department, which revealed a small hyperdense lesion in the right frontal region and traumatic subarachnoid hemorrhage. There were no radiological features of raised intracranial pressure (ICP) (Fig. 1).

**Figure 2.**
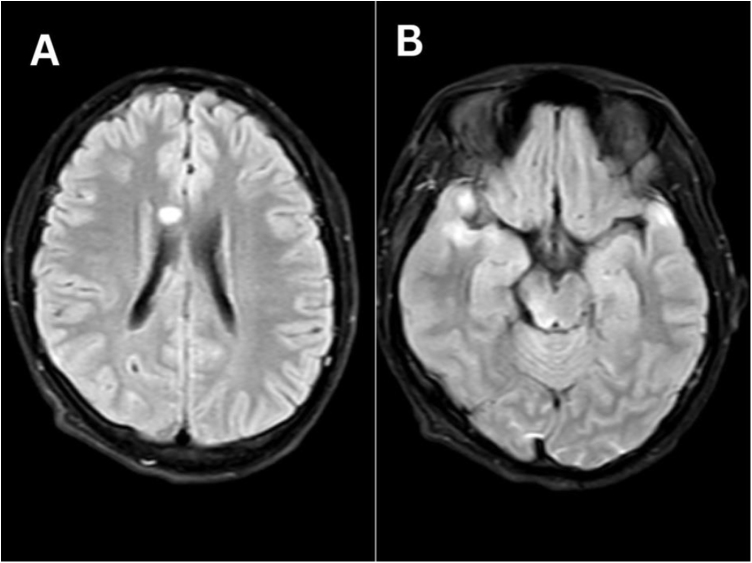
MRI brain, FLAIR, (A) showing hyperintense lesion in the corpus callosum, (B) showing hyperintense lesions in the dorsal aspect of the midbrain and cerebral cortex.

**Figure 3.**
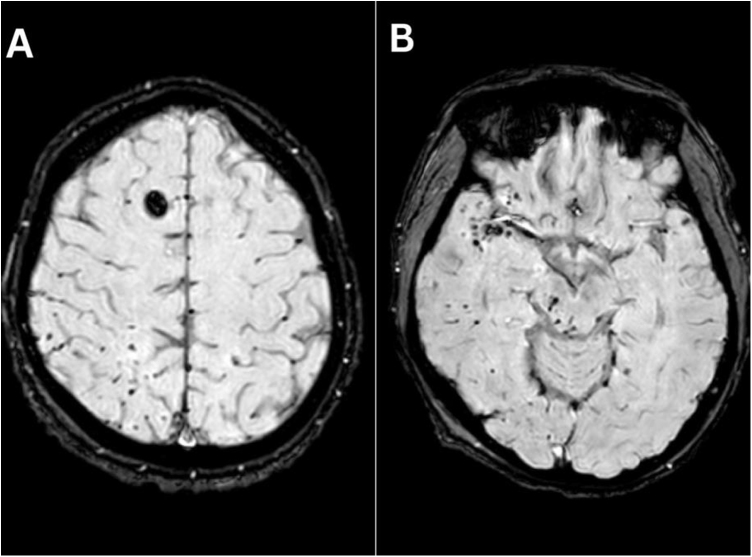
MRI brain, SWI, (A) showing multiple hypointense lesions throughout the cerebral cortex, (B) showing multiple hypointense lesions in the brainstem and cerebral cortex.

The patient was admitted to the neurosurgical intensive care unit (ICU) for observation and conservative management. During his initial ICU stay, his GCS fluctuated greatly between 10/15 and 15/15 with normal pupillary reaction. The CT head was repeated after 24 hours of admission, which was no different than his first CT scan. After 48 hours of admission, an MRI of the head was done, which was suggestive of severe DAI. MRI head revealed multiple punctate foci of altered signal intensity in bilateral cerebral hemispheres, genu of corpus callosum, and brainstem, which appeared isointense in T1 and hyperintense in T2 and FLAIR sequences (Fig. 2). On susceptibility weighted imaging (SWI), these areas showed hypointense blooming (Fig. 3). A few areas also illustrated restricted diffusion in diffusion weighted imaging (DWI) sequences.

Moreover, an ill-defined area of T1 iso and T2 hypointense signal intensity was noted in the gray-white matter junction in the right superior frontal lobe with surrounding edema. The corresponding area on SWI images showed hypointense blooming (Fig. 3). No restricted diffusion was noted in DWI sequences. This suggests contusion with surrounding edema in the right frontal lobe, which was also noted in his first CT-head.

After about 50 hours of admission, his GCS dropped to 3/15 with reactive and equal pupillary reaction. No abnormal body movements were recorded before his sensorium was altered. He was intubated and mechanically ventilated. Post-ventilation vitals and laboratory tests were normal. However, eventually, he showed features like tachycardia, hyperthermia, excessive sweating, spasticity of muscles, and excessive endotracheal tube secretions from the third day of admission, which continued till the eighth day. Investigations that were sent on the line of infection were unremarkable. Total blood counts and active phase reactants were within normal limits, and sputum culture was found to be negative. The symptoms were thought to be due to autonomic dysfunction, which got better with appropriate medical management. His GCS gradually improved, and he was extubated on the eighth day of admission with a GCS of 14 (E4V4M6). Post-extubation, slowness in motor and verbal responses was observed. During his ICU stay, he was treated with nimodipine, edaravone, modafinil, baclofen, and levetiracetam. His mild to moderate agitations were managed by intravenous haloperidol on a per-need basis. He was given prophylactic antibiotics, and all the other supportive factors like electrolytes, blood pressure, and nutrition were optimized. Chest and limb physiotherapy was done regularly.

Following continuous medical, physiotherapeutic, and supportive interventions, the patient made a gradual but steady recovery. After being hospitalized for 18 days, he was discharged with a GCS of 14 and was directed to the rehabilitation facility for further management. He was followed up every month after his discharge. He had mild difficulty in walking and slowed speech; however, other neurological examinations were unremarkable. He was advised to continue physiotherapy at every follow-up.

## Discussion

DAI was described as early as 1956 as a diffuse degeneration of cerebral white matter^[5,6]^. Over the years, various authors have referred to it using terms such as shearing injury, diffuse damage of immediate impact, diffuse white matter shearing injury, and inner cerebral trauma^[5,6]^. DAI is the major factor in determining morbidity and mortality in patients with traumatic brain injury (TBI)^[7]^. It is the most common cause of disability, post-traumatic coma, and persistent neurovegetative state^[7]^. Clinically, DAI can be defined as a state of coma (GCS<8) lasting for more than 6 hours, which is further classified into mild, moderate, and severe forms^[7,8]^. Mild DAI is characterized by a coma lasting up to 24 hours, with mild to moderate memory impairment and disability^[8]^. Moderate DAI refers to a coma that lasts longer than 24 hours and is associated with confusion, long-lasting amnesia, memory, and cognitive impairment^[8]^. Severe DAI involves a coma that can last for months, potentially resulting in flexor and extensor posturing, dysautonomia, cognitive defects, and impairment in memory, speech, and sensory-motor functions^[8]^.

Another widely used classification is the Adams classification of DAI, which categorizes this injury into three grades based on clinical presentations and the pathophysiological lesions found in the white matter tracts^[3]^. Grade 1 DAI is characterized by mild DAI with microscopic white matter changes in the cerebral cortex, corpus callosum, brain stem, and, less commonly, the cerebellum^[3,6]^. Grade 2 involves moderate DAI presenting with focal lesions in the corpus callosum. Grade 3 is classified as severe DAI, which includes focal lesions in the dorsolateral quadrant of the brainstem in addition to the changes noted in grade two^[3,6]^.

According to the Centers for Disease Control and Prevention (CDC), TBI-related deaths in the USA were over 69,000 in 2021, which accounted for 190 TBI-related deaths every day^[3,9]^. Although the true incidence of DAI is usually underestimated due to dilemmas in diagnosis and association with other traumatic brain injuries, such as subdural bleeding, epidural bleeding, and intracranial bleeding, it is roughly estimated that 10% of all patients with TBI may have some extent of DAI^[3]^. Death is inevitable in around 25% of patients with DAI^[3]^. The most common cause of DAI was found to be high-speed motor vehicle accidents (69%), followed by fall injury(18%)^[6]^. Our patient’s case highlights a significant concern, as their injury resulted from a fall, which also led to a right frontal lobe hemorrhage. This underscores the urgent need for preventive measures and awareness surrounding fall-related injuries.

The most common mechanism of DAI involves accelerating, decelerating, and rotational motion that leads to the shearing of white matter tracts of the brain, leading to microscopic and gross damage of axons in the junction of gray and white matter^[3,10]^. The differences in density between the cortex and white matter result in misaligned or stretched axons during closed head injuries. This leads to depolarization, metabolic alterations, cellular swelling, cytotoxic edema, and disruption of neuronal structure^[3,10]^. Additionally, the edema of axon or neuroglia is caused by activated cysteine protease, which destroys the cytoskeleton, leading to leakage of glutamate through the disordered membrane^[1]^. Research indicates that the late-stage clinical decline in DAI patients is less about immediate axonal injury and more about alterations in the axonal membrane, affecting the transport of crucial substances like cysteine and glutamate^[1]^.

In a study conducted in 1982, 45 cases of DAI were evaluated and compared with 132 cases of fatal head injury without DAI, which demonstrated the absence of lucid interval in DAI compared to other traumatic head injuries^[5]^. Lucid interval refers to whether the patient talked or responded after the impact or injury^[5]^. However, in a subsequent study, 17 out of 122 patients with DAI (14%) experienced a lucid interval or a delayed onset of symptoms^[6]^. Though DAI is commonly associated with an immediate loss of consciousness, a handful of cases of delayed-onset DAI have been reported^[1]^. The existing literature presents some interesting case reports detailing instances where patients experienced a loss of consciousness between 6 and 32 hours after sustaining trauma, underscoring the complexity and variability of DAI presentation and outcomes^[1,2,11,12]^.

Our patient presented with a history of a fall from the swing and was responsive for about 50 hours before his GCS dropped to < 8, necessitating intubation. The presence of bleeding in the frontal region, traumatic subarachnoid bleeding in the initial CT head, and unusual presentation overshadowed the diagnosis of DAI before the MRI was done. Subsequent evaluation of MRI findings and clinical presentation led to the diagnosis of delayed onset Grade 3 DAI. This highlights the importance of considering delayed onset DAI as a part of the primary differential diagnosis in patients with evolving neurologic symptoms following TBI, even in the absence of initial imaging findings.

The clinical picture of the patients with DAI varies significantly from minor concussive symptoms to profound loss of consciousness and even persistent vegetative state^[3]^. Typically, these patients require intubation and are admitted to the ICU for conservative management. Throughout the course of illness, a range of common features often emerge, including bradycardia, tachycardia, hypoxia, hyperglycemia, hypoglycemia, hypotension, and pupillary abnormalities^[7]^. One noteworthy and rare complication associated with DAI is dysautonomia, affecting approximately 10% of those surviving severe TBI with DAI^[13]^. Dysautonomia is classically characterized by tachycardia, tachypnoea, hyperthermia, hypertension, increased muscle tone, decerebrate or decorticate posturing, profuse sweating for at least three consecutive days, and absence of possible causes of infection^[13]^. In our patient, dysautonomia was evident on the third day of ICU admission. Thorough testing revealed negative results from sputum, blood, and urine cultures, effectively ruling out infection. These symptoms persisted for approximately 6 days but were managed effectively through symptomatic treatment.

It should be noted that when clinical findings in TBI are disproportionate to CT scan images, DAI should always be suspected^[11,14]^. Unfortunately, most imaging techniques tend to underestimate the extent of DAI^[11]^. The detection efficacy of CT scans for shearing injuries ranges from only 20% to 50%, whereas MRI is vastly superior in detecting these injuries^[11]^. A study on 24 patients with DAI highlighted that MRI is four times more effective than CT in detecting DAI^[15]^. Moreover, MRI excels at detecting non-hemorrhagic lesions, which constitute 75% of DAI cases^[15]^. Lesions associated with DAI are most commonly located in the lobar white matter (96%), followed by the corpus callosum (70%) and rostral brainstem (42%)^[15]^. MRI not only aids in detection but is also instrumental in classifying DAI into three distinct grades, taking into account lesion location, number, and severity^[15]^. For optimal imaging results, the ideal time to perform an MRI following a TBI is between 3 and 7 days after the incident^[11]^. The most frequent MRI finding is multifocal areas of brightness in the corpus callosum, which are typically appreciated in T2-weighted images^[11]^. While MRI is the commonly used imaging modality in our region, emerging studies highlight the remarkable potential of diffusion tensor imaging (DTI) in diagnosing DAI^[14]^.

The primary objective in managing patients with DAI is to provide comprehensive supportive care while actively preventing further secondary complications such as hypoxia, cerebral edema, and the detrimental effects of elevated ICP^[3]^. This can be accomplished through timely intervention, effective resuscitation, and vigilant monitoring of the patient’s condition^[3]^. For those with a GCS score below 8, it is crucial to consider intensive measures like ICP (ICP) monitoring and the potential need for intubation^[3]^. Furthermore, initiation of short-term treatment with anticonvulsants is vital to prevent early post-traumatic seizures^[3]^. It is important to recognize that a prolonged recovery can often be anticipated following DAI, necessitating a multi-faceted rehabilitation approach involving speech, occupational, and physical therapies, along with psychosocial support^[16]^.

In our case, the patient received conservative management at our institute along with the necessary supportive treatment and rehabilitation. Although the unusual presentation initially posed a diagnostic dilemma, the MRI findings and clinical features ultimately guided us to a definitive diagnosis. Delayed presentation of DAI, though uncommon, is a potential reality. Therefore, it is crucial to conduct a thorough assessment of every patient with TBI who may have DAI, even when initial CT scans appear normal. Increased awareness of the atypical presentations of DAI empowers clinicians to navigate diagnostic challenges effectively, ultimately enhancing patient care and outcomes.

This case also suggests a need for heightened clinical suspicion and perhaps more aggressive use of advanced imaging (e.g., DTI MRI) in patients with worsening symptoms despite initial normal scans^[14]^. Future research could explore biomarkers or imaging protocols that facilitate the early detection of delayed-onset DAI^[17]^.

## Conclusion

DAI should always be suspected when the clinical presentation of a patient is disproportionately more severe than the initial findings in CT head^[11]^. Though DAI typically leads to an immediate loss of consciousness, there have been rare cases of delayed presentation, as seen in our patient^[1]^. This highlights the importance of monitoring patients in medical facilities after major trauma and underscores the need for MRI to diagnose DAI in cases of delayed presentation. To address the challenges in diagnosing and treating delayed onset DAI, clinicians should maintain a high index of suspicion and implement a prompt management strategy that includes supportive care and rehabilitation. These interventions can significantly contribute to improving patient outcomes.

## Data Availability

None.
